# Dry eye management in a Sjögren’s syndrome mouse model by inhibition of p38-MAPK pathway

**DOI:** 10.1186/1746-1596-9-5

**Published:** 2014-01-20

**Authors:** Xiaoyun Ma, Jun Zou, Linping He, Yun Zhang

**Affiliations:** 1 Department of Ophthalmology, Guanghua Integrative Medicine Hospital, Shanghai, Changning District, China; 2 Department of Ophthalmology, Shanghai Jiao Tong University Affiliated Sixth People’s Hospital, Shanghai, China; 3 Institute of Arthritis Research, Shanghai Academy of Chinese Medical Sciences, Shanghai, China

**Keywords:** Sjögren’s syndrome, Dry eye, Interleukin-1β, p38-MAPK pathway

## Abstract

**Background:**

To investigate the therapeutic effect of p38-MAPK inhibitor, SB203580, on dry eye in a mouse model of Sjögren’s syndrome (MRL/lpr mice).

**Methods:**

18 female BALB/c mice and 44 female MRL/lpr mice were included. Mice were randomly assigned to the control or treatment group. The expression of phospho-p38 MAPK in lacrimal glands of BALB/c mice was determined by Western blot analysis following IL-1β treatment at various time points. Different doses of SB203580 were injected into lacrimal glands of MRL/lpr mice and phenol red thread test was performed seven days post-injection. Moreover, the levels of acetylcholine and norepinephrine expression in lacrimal glands of MRL/lpr mice were measured using spectrofluoremetric assay and the histopathology of lacrimal glands was also evaluated.

**Results:**

The expression of p-p38 MAPK in lacrimal glands of BALB/c mice gradually increased following incubation with IL-1β *ex vivo*. Injection of SB203580 into lacrimal glands significantly improved the results of phenol red thread test in MRL/lpr mice. In addition, the secretions of acetylcholine and norepinephrine increased significantly compared to the control group. Less lymphocytes infiltration was observed in pathological section of lacrimal glands following SB203580 injection.

**Conclusions:**

Our results indicate that the activation of p38-MAPK pathway plays an important role in dry eye of a Sjögren’s syndrome mouse model. Inhibition of p38-MAPK pathway by SB203580 might have potential therapeutic effect on Sjögren’s syndrome associated dry eye.

**Virtual slides:**

The virtual slides for this article can be found here: http://www.diagnosticpathology.diagnomx.eu/vs/1256849631103092.

## Introduction

The International Dry Eye WorkShop (DEWS) updated the definition of dry eye in 2007 [1], which reads as follows “*Dry eye is a multifactorial disease of the tears and ocular surface that results in symptoms of discomfort, visual disturbance, and tear film instability with potential damage to the ocular surface. It is accompanied by increased osmolarity of the tear film and inflammation of the ocular surface.*” Inflammation was especially highlighted in this new definition.

A deficiency in secretions of lacrimal and salivary glands is the primary cause of dry eye and dry mouth, and Sjögren’s syndrome is the leading cause of the aqueous tear-deficient dry eye [2,3]. Sjögren’s syndrome is an autoimmune disease that occurs almost exclusively in females (about 90%). This syndrome is associated with an extensive lymphocytic infiltration of the lacrimal and salivary glands and destruction of epithelial cells. To date there is no cure for this disease. Moreover, the exact cause of Sjögren’s syndrome is largely unknown but may involve numerous factors including those of viral, endocrine, neural, genetic, and environmental origin [4-6].

Reflexes from ocular surface and optic nerve, as well as from higher centers of the brain, stimulate lacrimal gland secretion through parasympathetic and sympathetic efferent pathways [7-9]. Parasympathetic and sympathetic nerves innervate the acinar cells, duct cells, and blood vessels of the lacrimal and salivary glands. The parasympathetic nerves contain the neurotransmitter acetylcholine, which acts through cholinergic muscarinic receptors, and vasoactive intestinal peptide. Sympathetic nerves contain norepinephrine, which acts through adrenergic receptors. The study of Zoukbri *et al.* showed that stimulation of nerves from inflamed, but not those from noninflamed, lacrimal and salivary glands with high concentration of KCl failed to increase the release of acetylcholine. Moreover, they also found that the activation of noninflamed lacrimal gland nerves with high KCl resulted in protein secretion whereas activation of inflamed glands did not. These findings demonstrate that, as suggested earlier by Sullivan, inflammation of exocrine glands in Sjögren’s syndrome results in impaired release of neurotransmitters from nerves, which leads to decreased fluid secretion.

Several studies have shown that suppression of acetylcholine and norepinephrine release from myenteric nerves was mediated by proinflammatory cytokines including interleukin (IL)-1β, IL-6, and tumor necrosis factor (TNF)-α [10-12]. IL-1β was implicated in blocking KCl-induced norepinephrine release from the myenteric plexus. IL-1β has also been shown to decrease the acetylcholine level in rat hippocampal formation. Zoukhri’s study [13] showed that the levels of proinflammatory cytokines were elevated in lacrimal and salivary glands of Sjögren’s syndrome patients as well as in animal models. Moreover, they found that the protein level of IL-1β was increased in the lacrimal and salivary glands of MRL/lpr mice which represents a mouse model of Sjögren’s syndrome in a disease-dependent manner.

The MRL/lpr mice and congenic MRL/Mp-lpr/lpr mice firstly described by Murphy were used as animal models to study another autoimmune disease, systemic lupus erythematosus. Later, it was found that these animals had coexisting Sjögren’s syndrome. NZB/NZW and MRL/lpr mice show spontaneous development of mononuclear cell infiltration of the salivary and lacrimal glands and other organs. In both animals, this disease occurs almost exclusively in females and progresses in an age-dependent manner. MRL/lpr mice, compared to NZB/NZW mice, have more pronounced and destructive mononuclear infiltrations in lacrimal and salivary glands [14].

The p38 mitogen-activated protein kinase (MAPK) pathway has been shown to be activated by IL-1β treatment in a number of cell types including lacrimal gland cells [15]. In this study, consistent with previous observation, we found that *ex vivo* incubation of normal lacrimal glands from BALB/c mice with IL-1β could activate the p38 MAPK pathway. We report here that administration of p38 MAP kinase inhibitor SB203580 in lacrimal glands of a Sjögren’s syndrome mouse model significantly alleviates the dry eye symptom, suggesting the potential clinical implication of SB203580 in the treatment of dry eye in Sjögren’s syndrome.

## Material and methods

### Animals

18 female BALB/c mice (15–20 weeks old) and 44 female MRL/lpr mice (18 weeks old, SPF) were purchase from Shanghai Laboratory Animal Center, Chinese Academy of Sciences. They were maintained in constant temperature rooms with fixed light–dark intervals of 12 hours’ length. All experiments were approved by the Research Ethics Board of Shanghai Jiao Tong University Affiliated Sixth People’s Hospital and Shanghai Guanghua Integrative Medicine Hospital and performed in accordance with the ARVO Statement for the Use of Animals in Ophthalmic and Vision Research.

### Chemicals

Acetylcholine assay kit, SB203580, recombinant mouse IL-1β, Krebs-ringer bicarbonate buffer (KRB) were purchased from Sigma (St. Louis, MO), Phospho-p38 MAP Kinase antibody was purchased from Cell Signaling Technology. Norepinephrine assay kit was ordered from Alpco.

### Western blot analysis of phospho-p38 MAPK in lacrimal glands

Lacrimal glands were removed from 15-20-week-old BALB/c. Tissue was cut into small lobules (2 mm in diameter), and incubated at 37°C in KRB buffer (pH 7.4) containing 10 ng/ml IL-1β for 0, 5, 10, 30, 60 and 120 min. Lobules were subjected to gentle pipetting through tips of decreasing diameter. The preparation was then filtered through nylon mesh (150 μm), and the acini were pelleted by centrifugation (50 g, 2 min). The pellet was washed through KRB containing 4% BSA by centrifugation (50 g, 2 min). To remove lymphocytes, acini were subjected to a Ficoll gradient of 2%, 3%, and 4%. Dispersed acini were allowed to recover for 30 min in fresh KRB buffer containing 0.5% BSA, after which they were homogenized in 0.3 mL of 10 mM Tris–HCl (pH 7.0). For Western blot analyses, equal amounts of total protein from acinar cell lysates were separated on 10% sodium dodecyl sulfate-polyacrylamide gels, followed by electrotransferred to nitrocellulose membranes. The phospho-p38 protein was detected by the p-p38 antibody (dilution, 1:1000).

### Injection of SB203580 into lacrimal glands

Isoflurane was used to anesthetize MRL/lpr mice. SB203580 (25 μM, 250 μM, 2.5 mM) or saline in a total volume of 2 μl was injected into the exorbital lacrimal glands of anesthetized mice. Injections were performed once a day for 7 consecutive days [16].

### Phenol red thread test, break up time (BUT) test and fluorescein staining

**Figure 1:**
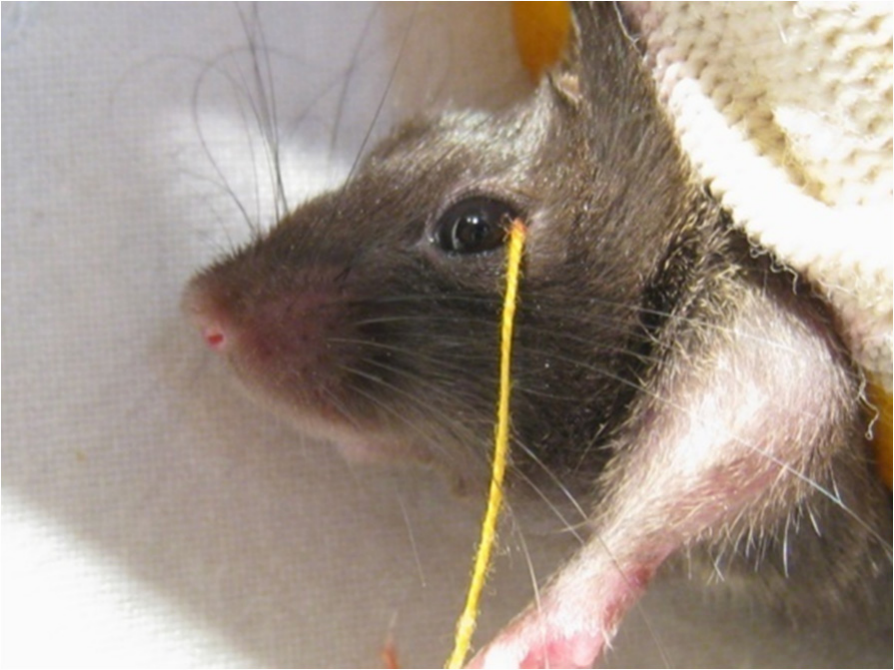
Tear production was measured on lightly anesthetized mice using phenol red impregnated cotton threads.

After SB203580 injection into lacrimal glands of MRL/lpr mice for seven days, phenol red thread test, tear BUT and fluorescein staining were performed to determine tear secretion and stability of tear film. Tear production was measured in lightly anesthetized mice using phenol red impregnated cotton threads (Zone-Quick, Lacrimedics). The threads were held with jeweler forceps and applied to the lateral canthus of both eyes for 10 seconds (Figure 1). Wetting of the thread (which turns red in contact with tears) was measured in millimeters under a dissecting microscope [16]. 0.5 μl of 5% fluorescein sodium was applied to the murine conjunctival sac. BUT and corneal staining were observed by slit lamp microscope. The staining indicates damage of corneal epithelium. The staining grade was classified by the standard: Grade 0: no staining; grade 1: 1/8 was stained; grade 2: 1/4 was stained; grade 3: 1/2 was stained; grade 4: > 1/2 was stained.

### Assay of acetylcholine and norepinephrine

The amount of acetylcholine and norepinephrine were measured using the acetylcholine assay kit (Amplex Red; Molecular Probes) and norepinephrine assay kit (Alpco, NH, US) according to manufactory’s instruction. This kit measures the amount of hydrogen peroxide (which in the presence of horseradish peroxidase leads to the oxidation of Amplex Red) produced through the oxidation of choline. For the measurement of acetylcholine, 0.1 ml of media and tissue homogenate were spotted in duplicate into 96-well plates. An acetylcholine standard curve was used in each experiment. In each well, 0.1 ml of assay buffer (50 mM Tris–HCl, pH 7.5) containing 0.2 M Amplex Red reagent, 2 U/ml horseradish peroxidase, 0.2 U/ml choline oxidase, and 10 U/ml acetylcholinesterase was added. After incubation, the fluorescence was determined in a fluorescence microplate reader (model FL600; Bio-Tek, Winooski, VT) using 530 nm excitation wavelength. The concentration of acetylcholine was determined using the software provided by the manufacturer (KC4; Bio-Tek). For the measurement of norepinephrine, Pipette 100 μL of samples including standards and controls from the Enzyme Plate into the respective pre-coated norepinephrine microtiter strips. Then pipette 50 μL of the respective norepinephrine antiserum into all wells, cover the plate with adhesive foil. Incubate for 1 min at room temperature (20-25°C) on a shaker. After incubation for 15 to 20 hours at 4°C, remove the foil and discard the contents in the wells and wash each well 4 times thoroughly with 300 μL wash buffer. Blot dry by tapping the inverted plate on absorbent material. Pipette 100 μL of Enzyme Conjugate into all wells. Then cover the plate with Adhesive Foil and incubate 30 min at room temperature (20-25°C) on a shaker. Remove the foil and discard the contents of the wells and wash each well 4 times thoroughly with 300 μl wash buffer. Blot dry by tapping the inverted plate on absorbent material. Pipette 100 μl of substrate into all wells, and incubate 20–30 min at room temperature (20-25°C) on a shaker. Pipette 100 μl of stop solution into all wells. Finally read the absorbance of the solution in the wells within 10 min, using a microplate reader set to 450 nm and a reference wavelength between 620 nm and 650 nm [14].

### Lacrimal glands histopathology

Lacrimal glands pieces were fixed in 4% paraformaldehyde for 24 hours. After incubation in 30% sucrose overnight, the tissue was frozen in O.C.T. embedding medium. Cryostat sections (6 μm) were placed on gelatin-coated slides and dried overnight at 37°C. For histopathology experiments, sections were stained with hematoxylin and eosin.

### Statistical analysis

The data are presented as mean ± standard deviation (SD) and number (n). One way analysis of variance (ANOVA) test was used followed by post hoc test to determine the significance of variables when comparing more than 2 groups. Statistical significance is considered a value of *P* <0.05. All statistical analyses were performed using SPSS software, version 10.0.

## Results

### Effects of IL-1β on p38-MAPK activity in lacrimal glands of BALB/c mice

**Figure 2:**
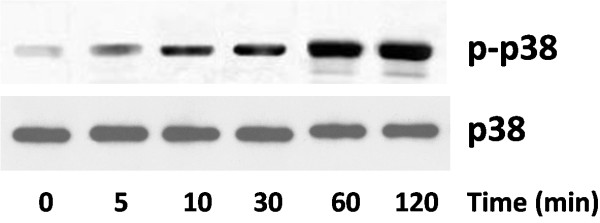
**Effects of IL-1β on p38 MAPK activity in lacrimal gland of BALB/c mice.** Western blot analysis of p-p38 MAPK in lacrimal gland of BALB/c mice treated with IL-1β (10 ng/ml). The experiment was performed in duplicate with samples from three mice for each time point.

Lobules were prepared from lacrimal glands of female BALB/c mice and incubated for 0–120 minutes (0, 5, 10, 30, 60, and 120 min) with or without IL-1β (10 ng/ml). p38 MAPK activity was determined by western blotting using an antibody that specifically recognizes the phosphorylated form of p38 MAPK. As shown in Figure 2, IL-1β induced a time-dependent activation of p38 MAPK.

### Effect of blocking p38 MAPK activity on tear production

**Figure 3:**
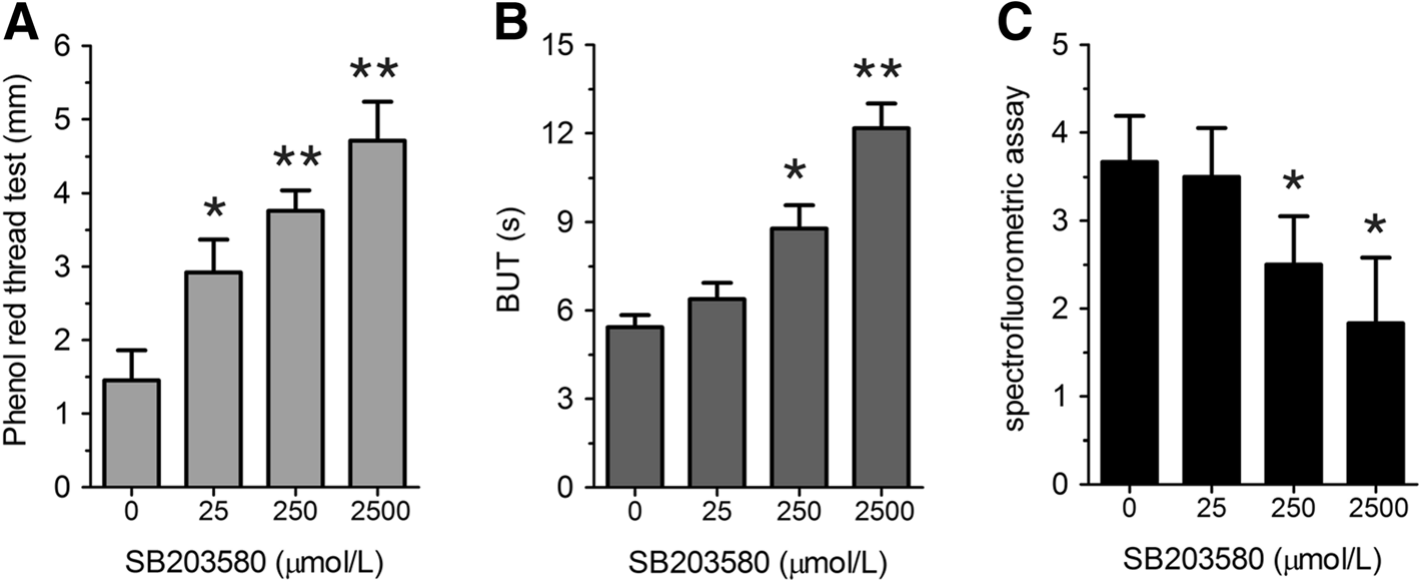
**Effect of lacrimal gland injection of SB203580 on tear production. (A-C)** Phenol red thread test **(A)**, tear break up time (BUT) **(B)**, and spectrofluorometric assay **(C)** were performed on MRL/lpr mice after indicated concentrations of SB203580 were injected for seven days. (n = 8) *P < 0.05, **P < 0.01 compared to controls.

Female MRL/lpr mice spontaneously develop, in an age-dependent manner, an autoimmune disease characterized by lymphoproliferation, autoantibody formation, ocular inflammatory lesions, and lacrimal gland disease and has been widely used as a research model for human Sjögren’s syndrome dry eye. As mentioned above, the protein level of IL-1β increased in lacrimal and salivary glands of MRL/lpr mice, we next tested whether blocking IL-1β could modify the disease phenotype. We found that injection with SB203580, a p38-MAPK pathway inhibitor, significantly increased the tear production compared to the vehicle (PBS) injected group (Figure 3A,B). In parallel studies, we confirmed that there was no difference in tear production in the vehicle-injected group compared to the non-injected group. Furthermore, the corneal epithelium damage by inflammation was alleviated shown by significant decrease of staining grade (Figure 3C).

### Blocking p38 MAPK pathway increased the amount of acetylcholine and norepinephrine

**Figure 4:**
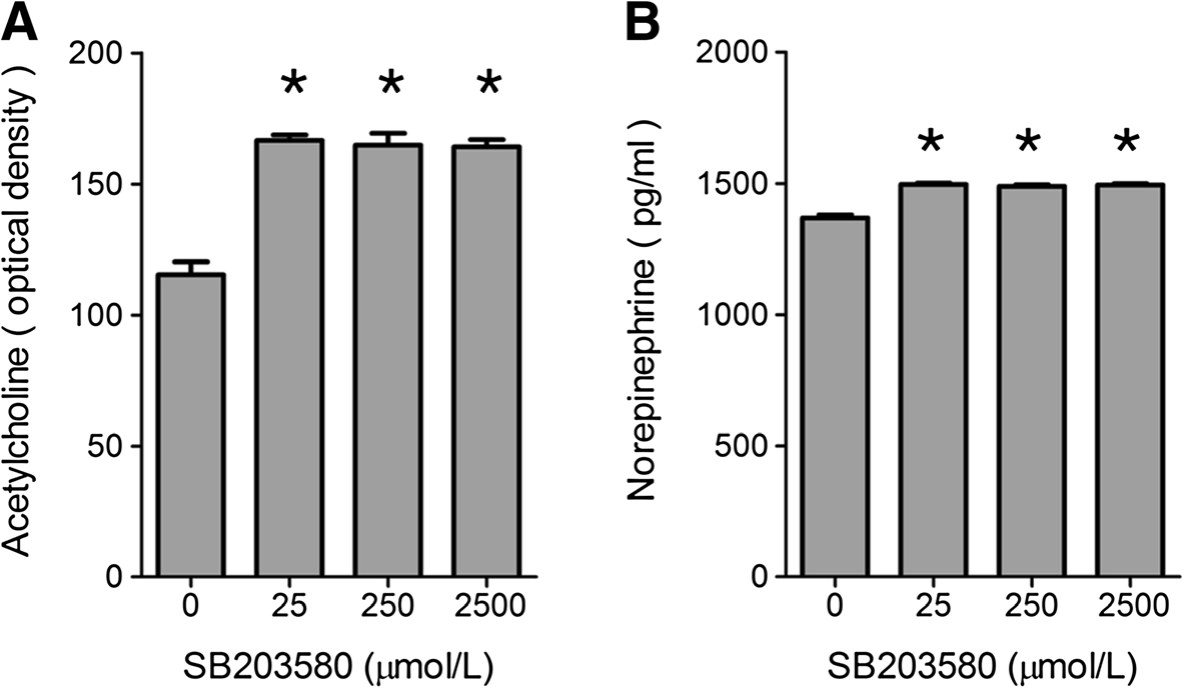
**Acetylcholine and norepinephrine secretion in different groups. (A,B)** Acetylcholine **(A)** and norepinephrine **(B)** assays were performed on MRL/lpr mice after indicated concentrations of SB203580 were injected for seven days. (n = 8) *P < 0.05 compared to controls.

Secretion of acetylcholine and norepinephrine was detected in lacrimal gland tissue in PBS and SB203580 injection groups. As shown in Figure 4, both acetylcholine and norepinephrine secretion increased significantly after SB203580 injection for 7 days, but not dose dependently.

### Lacrimal glands histopathology

**Figure 5:**
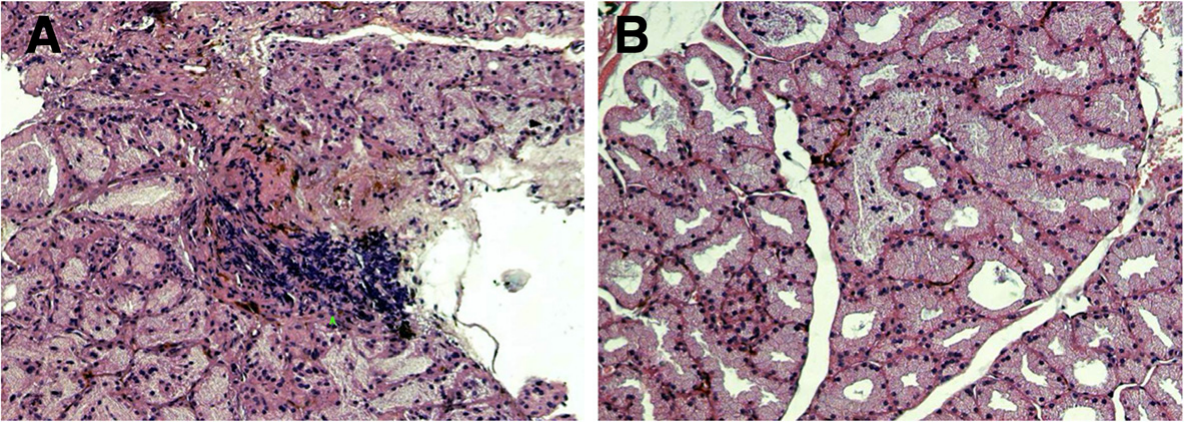
**Lacrimal gland histopathology of MRL/lpr mice with or without SB203580 injection (hematoxylin eosin staining, ×100). A**, Lacrimal gland from PBS injection group; **B**, Lacrimal gland from 2500 μmol/L SB203580 injection group.

In PBS injection group, large quantities of lymphocyte infiltration were shown, there was a severe inflammatory response with inflammatory cells invading the interlobular space and surrounding both acinar and ductal cells (Figure 5A). After injection different concentration of SB203580, There was much less inflammation (Figure 5B).

## Discussion

Nowadays the therapies for dry eye in clinic mainly focus on remission of symptoms, but not aiming at blocking pathogenesis of this disease [17]. Though cyclosporine eye drops and steroid hormone which could inhibit immune response has been developed for the treatment of dry eye, it could induce impairment of ocular surface defense barrier, secondary infection, and tear secretion. In this study, we investigated the signal transduction pathway through which IL-1β inhibited neurotransmitter release from lacrimal gland nerves of MRL/lpr mice, a murine model of Sjögren’s syndrome. We also tested the therapeutic effect of p38 MAPK inhibitor SB203580 injection on Sjögren’s syndrome dry eye, hoping for developing new management method in clinic.

Sjögren’s syndrome, together with systemic lupus erythematosus (SLE), scleroderma, *et al.*, are so-called autoimmune connective tissue diseases, characterized by presence of antinuclear antibodies (ANA) in the blood of patients [18]. A histological hallmark of SLE is membranous glomerulonephritis, due to immune complex deposition along the glomerular basement membrane. Scleroderma is characterized by progressive tissue fibrosis, with skin most commonly affected by edema and perivascular CD4+ and CD8+ lymphocytic infiltrations. Sjögren’s syndrome is characterized by decreased secretion of tears and saliva caused by inflammation of lacrimal and salivary glands. These diseases are most frequent systemic autoimmune diseases, occurring under a condition in which innate tolerance is damaged [19].

MRL/lpr mice develop spontaneous autoimmune disease during aging, which is characterized by lymphocyte proliferation, autoantibody formation, ocular surface inflammation and lacrimal gland inflammation [14]. It is an animal model widely used in the study of Sjögren’s syndrome. Large amount of IL-1β could be detected in the lacrimal gland of MRL/lpr mice, and the level increases with age, we found that *ex vivo* incubation of lacrimal glands isolated from healthy BALB/c mice with IL-1β resulted in the activated p38MAPK in a time-dependent manner, suggesting the role of p38 MAPK pathway in IL-1β-induced inflammation of lacrimal glands.

To test the role of p38 MAPK *in vivo*, we injected p38 MAPK inhibitor SB203580 into lacrimal gland of MRL/lpr mice. We found that seven days injection of p38MAPK inhibitor can significantly improved the disease phenotype in MRL/lpr mouse model of Sjögren’s syndrome including increased tear production. This improvement coincides with increased secretion of neurotransmitters acetylcholine and norepinephrine and reduced infiltration of inflammatory cells.

In conclusion, in this study, we investigated the role of the p38MAPK signal transduction pathway in inhibition of neurotransmitter secretion in lacrimal gland. We demonstrated the preclinical efficacy of p38-MAPK inhibitor SB203580 on lacrimal gland secretion and neurotransmitter secretion. Our study strongly suggests that SB203580 can potentially further developed to disease-modifying agent to prevent and/or treat Sjögren’s syndrome dry eye.

## Competing interests

The authors declare that they have no competing interest.

## Authors’ contributions

XM and JZ constructed the manuscript. YZ and LH performed the western blot analysis. XM and YZ carried out the animal study and Ophthalmology test study. JZ and YZ designed and constructed manuscript. All authors read and approval the final manuscript.
